# Next generation of immune checkpoint therapy in cancer: new developments and challenges

**DOI:** 10.1186/s13045-018-0582-8

**Published:** 2018-03-15

**Authors:** Julian A. Marin-Acevedo, Bhagirathbhai Dholaria, Aixa E. Soyano, Keith L. Knutson, Saranya Chumsri, Yanyan Lou

**Affiliations:** 1 Department of Internal Medicine, Mayo Clinic, Jacksonville, FL USA; 2 Division of Hematology and Oncology, Mayo Clinic, Jacksonville, FL USA; 3 Present Address: Department of Blood and Marrow Transplantation and Cellular Immunotherapy, Moffitt Cancer Center, Tampa, FL USA; 4 Division of Immunology, Mayo Clinic, Jacksonville, FL USA

**Keywords:** Cancer, Immunotherapy, Tumor microenvironment, Immune evasion, Cytotoxic T lymphocytes, Immunotherapy, Immune checkpoint therapy, Co-stimulatory pathways, Inhibitory pathways, Tumor microenvironment

## Abstract

Immune checkpoints consist of inhibitory and stimulatory pathways that maintain self-tolerance and assist with immune response. In cancer, immune checkpoint pathways are often activated to inhibit the nascent anti-tumor immune response. Immune checkpoint therapies act by blocking or stimulating these pathways and enhance the body’s immunological activity against tumors. Cytotoxic T lymphocyte-associated molecule-4 (CTLA-4), programmed cell death receptor-1 (PD-1), and programmed cell death ligand-1(PD-L1) are the most widely studied and recognized inhibitory checkpoint pathways. Drugs blocking these pathways are currently utilized for a wide variety of malignancies and have demonstrated durable clinical activities in a subset of cancer patients. This approach is rapidly extending beyond CTLA-4 and PD-1/PD-L1. New inhibitory pathways are under investigation, and drugs blocking LAG-3, TIM-3, TIGIT, VISTA, or B7/H3 are being investigated. Furthermore, agonists of stimulatory checkpoint pathways such as OX40, ICOS, GITR, 4-1BB, CD40, or molecules targeting tumor microenvironment components like IDO or TLR are under investigation. In this article, we have provided a comprehensive review of immune checkpoint pathways involved in cancer immunotherapy, and discuss their mechanisms and the therapeutic interventions currently under investigation in phase I/II clinical trials. We also reviewed the limitations, toxicities, and challenges and outline the possible future research directions.

## Background

**Fig. 1:**
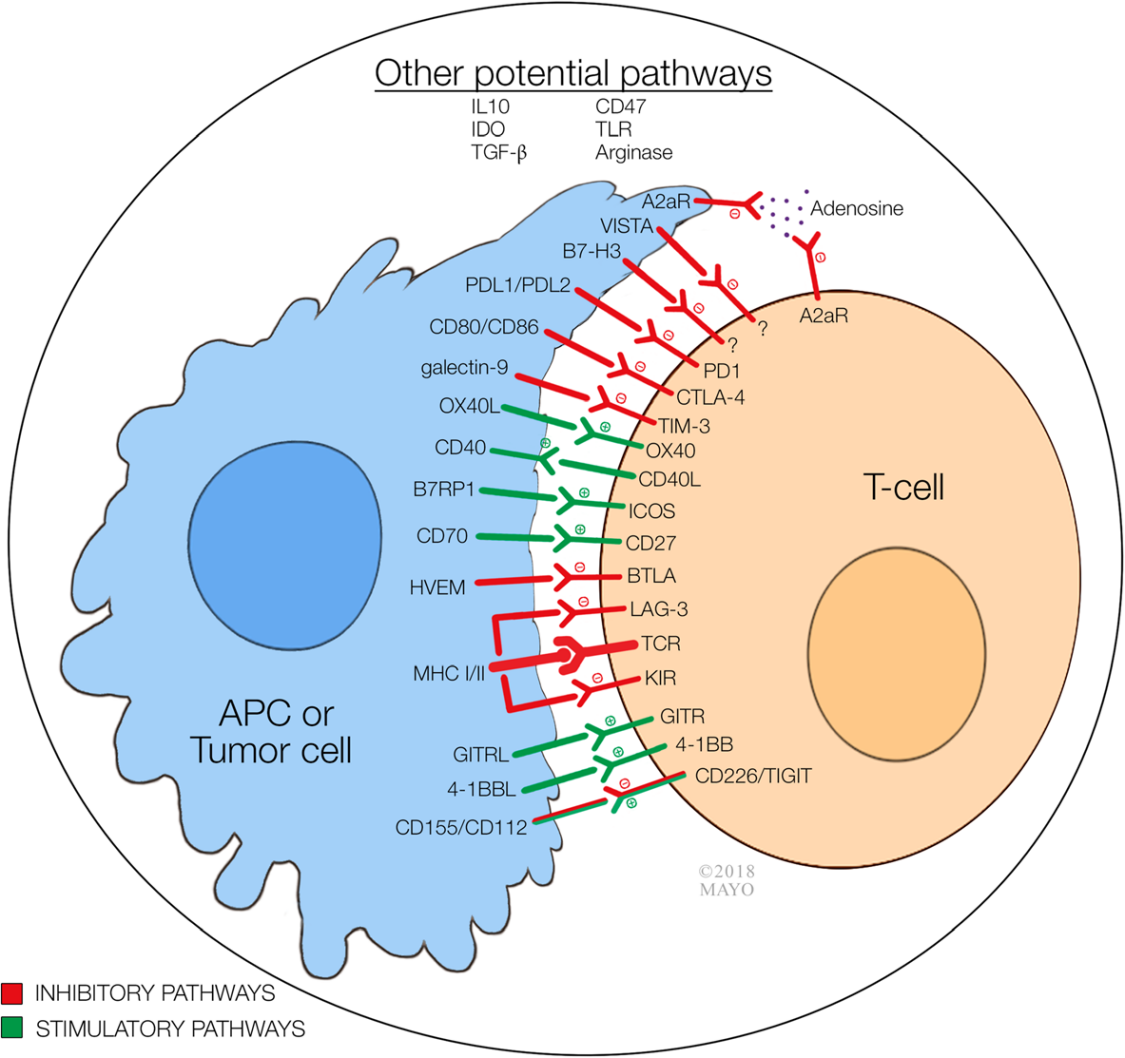
Immune interactions involving antigen presenting cells or tumor cells, T cells, and tumor microenvironment

Tumor immune micro-environment encompasses a wide range of complex interactions between tumor cell, immune cells (antigen presenting cells, T cell, NK cell, B cell, etc.), and tumor stroma. Host immune response against tumor is a result of competition between inhibitory and stimulatory signals. Immune checkpoints are important immune regulators in maintaining immune homeostasis and preventing autoimmunity. These consist of both stimulatory and inhibitory pathways that are important for maintaining self-tolerance and regulating the type, magnitude, and duration of the immune response. Under normal circumstances, immune checkpoints allow the immune system to respond against infection and malignancy while protecting tissues from any harm that may derive from this action. However, the expression of some of these immune-checkpoint proteins by malignant cells dysregulates the antitumor immunity and favors the growth and expansion of cancer cells [1]. Figure 1 summarizes these molecules and their targets [1–3]. Immune checkpoint therapy for cancer encompasses strategies that target these regulatory pathways in order to enhance immunity activity against tumor cells [4, 5]. The most broadly studied checkpoints are the inhibitory pathways consisting of cytotoxic T lymphocyte-associated molecule-4 (CTLA-4), programmed cell death receptor-1 (PD-1), and programmed cell death ligand-1 (PD-L1). Ipilimumab [anti-CTLA-4 monoclonal antibody (mAb)] was the first immune checkpoint inhibitor (ICI) approved by FDA in 2011 [6]. Many biological agents that target these molecules are now broadly used in a variety of malignancies. Currently approved ICIs are only effective in a small fraction of patients and resistance after initial response is a common phenomenon. Nevertheless, new inhibitory and stimulatory pathways have emerged as potential targets for immune checkpoint therapy and immunotherapy is extending even beyond this approach [7, 8]. Novel immune checkpoint agents and combination therapies currently under investigation in phase I/II clinical trials are reviewed and discussed in this article.

## Methodology

We performed a PubMed search using the keywords and MeSH terms immunotherapy, immune checkpoint therapy, immune checkpoint inhibitors, immune checkpoint agonists, and immune checkpoint adjuvants. We also searched American Society of Clinical Oncology (ASCO) and American Association for Cancer Research (AACR) meeting abstracts, and ClinicalTrials.gov from June 5, 2016, through January 30, 2018. We focused on phase I and phase II clinical trials of new agents in immune checkpoint therapy that were being used alone or in conjunction with other forms of immunotherapy. Data was collected from the trials reviewed with at least preliminary results published or presented before the date of the search. Exclusion criteria included phase III or later stage clinical trials, clinical trials focusing only on anti-CTLA-4 and anti-PD-1/PD-L1, trials focusing on pediatric population, and non-interventional trials. We have included 62 phase I and 23 phase II clinical trials in this review. Table 1 summarizes these agents and clinical trials.

**Table 1:** Summary of ongoing phase 1/2 clinical trials utilizing immune checkpoint therapy

Category	Target	Drug	Trial	Phase	Type of tumor	Clinical efficacy	Safety	Comments
Inhibitory pathways	LAG-3 (CD223)	IMP321 (Immuntep®)	NCT00732082	I	Pancreatic cancer	No OR	1/17 rash, no severe AEs	In combination with gemcitabine
			NCT00349934	I	BC	ORR 50%	No significant AEs from IMP321 alone	In combination with paclitaxel
			NCT02614833	II	BC	–	–	Ongoing
		BMS-986016	NCT01968109	I	Melanoma	ORR 16%, DCR 45%	Similar to nivolumab alone	In combination with nivolumab
		LAG525	NCT02460224	I/II	Solid malignancies	–	–	Ongoing
	TIM-3	MBG453	NCT02608268	I/II	Advanced malignancies	–	–	Ongoing
		MEDI9447	NCT02503774	I	Solid tumors	–	–	Ongoing
	TIGIT	OMP-31M32	NCT03119428	I	Solid tumors	–	–	Ongoing
	VISTA	JNJ-61610588	NCT02671955	I	Advanced tumors	–	–	Ongoing
		CA-170	NCT02671955	I	Solid tumors and lymphomas	–	–	This molecules also inhibits PD-L1/PD-L2
	B7-H3 (CD276)	Enoblituzumab (MGA271)	NCT01391143	I	Melanoma, prostate, solid tumors	SD > 12 weeks and tumor shrinkage (2–69%) in different tumor types	No dose-limiting toxicities, 6% with grade 3 AEs	Ongoing
			NCT02475213	I	Melanoma, HNSCC, NSCLC, urothelial cancer	–	–	In combination with pembrolizumab
		MGD009	NCT02628535	I	Melanoma, NSCLC, mesothelioma, urothelial cancer	–	–	DART protein that binds both CD3 and B7-H3
		8H9	NCT00089245	I	Neuroblastoma	17/21 patients were alive and free of disease after a median follow-up of 33 months	Self-limited myelosuppression	Antibody labeled with radioactive iodine
			NCT01099644	I	8H9-positive solid tumor involving peritoneum	–	–	Ongoing
			NCT01502917	I	Gliomas	–	–	Ongoing
			NCT00089245	I	CNS malignancies	–	–	Ongoing
	KIR	Lirilumab	NCT01714739	I/II	HNSCC	ORR 24%, DCR 52%	15% with grade 3–4 AEs	In combination with nivolumab and ipilimumab
		IPH4102	NCT02593045	I	CTCL	ORR 45%, 10/22 patients PR, 2 CR in skin and 5 CR in blood	6/22 with grade 3 or more AEs	Ongoing
	A2aR	CPI-444	NCT02655822	I	Solid tumors	DCR 42%	Most were mild, only 1/24 grade 3 AE (autoimmune hemolytic anemia)	Alone and in combination with atezolizumab
	TGF- β	Trabedersen (AP12009)	NCT00844064	I	Pancreatic cancer	Improved OS by 9.9–11.8 months, no improvement in PFS	-	Synthetic antisense oligonucleotide that hybridizes with RNA
		M7824	NCT02517398	I	Solid tumors	1/16 patients CR, 1 PR, 2 SD, 1 with 25% reduction of lesion size	No grade 4–5 AEs	Dual anti-PD-L1 antibody with TGF-β trap
		Galusertinib (LY2157299)	NCT01582269	II	Glioblastoma	No OS difference compared to lomustine alone	54% with grade 3–4 AEs	Alone or in conjunction with lomustine
			NCT02423343	I/II	NSLC, HCC	–	–	Ongoing
			NCT02734160	I	Pancreatic cancer	–	–	Ongoing
			NCT02672475	I	BC	–	–	Ongoing
	PI3Kγ	IPI-549	NCT02637531	I	Melanoma, NSCLC, HNSCC	12/15 patients with durable clinical benefit	No dose-limiting toxicities	Monotherapy or in conjunction with nivolumab
	CD47	Hu5F9-G4	NCT02216409	I	Solid tumors	2/16 patients SD for 8–16 months	Anemia 11/16 patients, hyperbilirubinemia 5/16, retinal toxicity 1/16	Ongoing
			NCT02953509	I/II	Relapsed or refractory NHL	–	–	In conjunction with rituximab Ongoing
		TTI-621 (SIRPαFc)	NCT02890368	I	Relapsed or refractory solid tumors and mycosis fungoides	–	–	Ongoing
	CD73	MEDI9447	NCT02503774	I	Solid tumors	–	–	Ongoing
Stimulatory pathways	OX40	9B12	NCT01644968	I	Solid tumors	12/30 patients with tumor regression but not achieving PR, 6/30 SD	7/30 patients with grade 3 or more lymphopenia	Completed
		MOXR 0916	NCT02410512	I	Solid tumors	–	No dose-limiting toxicities	In conjunction with atezolizumab
		PF-04518600 (PF-8600)	NCT02315066	I	Melanoma, NSCLC	4/9 patients SD	No dose-limiting toxicities or grade 3–5 AEs	Ongoing
		MEDI6383	NCT02221960	I	Solid tumors	–	–	Ongoing
		MEDI0562	NCT02705482	I	Solid tumors	–	–	Ongoing
		INCAGN01949	NCT02923349	I	Solid tumors	–	–	Ongoing
		GSK3174998	NCT02528357	I	Solid tumors	–	–	Ongoing
	GITR	TRX-518	NCT01239134	I	Solid tumors	4/40 patients SD	No dose-limiting toxicities or grade 3–5 AEs	Ongoing
		BMS-986156	NCT02598960	I	Solid tumors	–	1/66 patients with grade 4 creatine phosphokinase elevation leading to discontinuation of treatment	Alone or in conjunction with nivolumab
		AMG 228	NCT02437916	I	CRC, HNSCC, urothelial carcinoma, and melanoma	0/30 patients had OR	27/30 had AEs consisting of hypophosphatemia, anemia, and fever	Terminated (business decision)
		MEDI1873	NCT02583165	I	Solid tumors	–	–	Ongoing
		MEDI6469	NCT02559024	I	CRC	–	–	Ongoing
		MK-4166	NCT02132754	I	Solid tumors	–	–	Ongoing
		INCAGN01876	NCT02697591	I/II	Solid tumors	–	–	Ongoing
			NCT03126110	I/II	Solid tumors	–	–	Alone or in conjunction with nivolumab or ipilimumab. Ongoing
		GWN323	NCT02740270	I	Solid tumors and lymphomas	–	–	Ongoing
	ICOS	JTX-2011	NCT02904226	I/II	Solid tumors	Not reported	No dose-limiting toxicities, 3/25 patients with grade 3 AEs	Alone or in conjunction with nivolumab. Ongoing
		GSK3359609	NCT02723955	I	Solid tumors	–	–	Ongoing
		MEDI-570	NCT02520791	I	NHL	–	–	Ongoing
	4-1BB (CD137)	Utomilumab (PF-05082566)	NCT02179918	I	Solid tumors	6/23 patients CR or PR	No dose-limiting toxicities, most were grade 1–2 AEs	In combination with pembrolizumab
			NCT01307267	I	NHL, NSCLC, RCC, HNSCC, melanoma	–	–	Alone or in conjunction with rituximab. Ongoing
			NCT02444793	I	Solid tumors	–	–	In conjunction with mogamulizumab
			NCT02315066	I	HNSCC, HCC, melanoma, RCC	–	–	In conjunction with OX40 Agonist PF-04518600
			NCT02554812	II	Solid tumors	–	–	In conjunction with avelumab
		Urelumab	NCT02253992	I/II	Solid tumors and NHL	3/60 patients with NHL achieved PR and 3/60 CR. 9/86 patients with combination therapy achieved PR	3% with elevated AST, 3% elevated ALT, 7% with serious AEs	In conjunction with nivolumab
			NCT01471210	I	Solid tumors and NHL	–	–	Completed
	CD27-CD70	ARGX-110	NCT01813539	I	T cell lymphoma	2/9 patients had a reduction of malignant clones > 90%, 1 radiological PR and 2 skin PR	–	Ongoing
			NCT01813539	I/II	CD70 positive malignancies	–	–	Ongoing
		BMS-936561 (MDX-1203)	NCT00944905	I	RCC and B cell lymphoma	SD 69%	2/16 patients had grade 3 hypersensitivity	Completed
		Varilumab	NCT02335918	I	Solid tumors	Not reported	1/33 patients developed a dose-limiting toxicity (hepatitis and kidney injury)	In conjunction with nivolumab
			NCT02924038	I	Gliomas	–	–	Ongoing
			NCT02302339	II	Melanoma	–	–	Ongoing
			NCT02386111	I/II	RCC	–	–	Terminated (portfolio re-prioritization)
			NCT02543645	I/II	Solid tumors	–	–	Terminated (portfolio re-prioritization)
	CD40	CP-870893	NCT01103635	I	Melanoma	–	–	Ongoing
			NCT02304393	I	Solid tumors	–	–	Ongoing
		APX005M	NCT02482168	I	NSCLC, melanoma, urothelial cancer, HNSCC	–	–	Ongoing
			NCT03165994	II	Esophageal and gastroesophageal tumors	–	–	Ongoing
			NCT02706353	I/II	Melanoma	–	–	In conjunction with pembrolizumab. Ongoing
			NCT03123783	I/II	Melanoma, NSCLC	–	–	In conjunction with nivolumab. Ongoing
		ADC-1013	NCT02379741	I	Solid Tumors	–	–	Completed
			NCT02829099	I	Solid Tumors	–	–	Ongoing
		JNJ-64457107	NCT02829099	I	Solid tumors	–	–	Ongoing
		SEA-CD40	NCT02829099	I	Solid tumors and lymphomas	–	–	Alone or in conjunction with pembrolizumab
		RO7009789	NCT02304393	I	Solid tumors	–	–	Ongoing
			NCT02588443	I	Pancreatic cancer	–	–	In conjunction with Nab-paclitaxel and gemcitabine. Ongoing
			NCT02760797	I	Solid Tumors	–	–	In conjunction with Emactuzumab. Ongoing
			NCT02665416	I	Solid Tumors	–	–	In conjunction with vanucizumab. Ongoing
Other pathways	IDO	BMS-986205	NCT02658890	I	Solid tumors	–	3/42 patients with grade 3 autoimmune hepatitis	In conjunction with nivolumab
		Indoximod	NCT02073123	II	Melanoma	ORR 52%	No significant toxicities	Used in conjunction with ipilimumab, nivolumab, or pembrolizumab
			NCT02077881	I/II	Pancreatic cancer	ORR 37%	1/30 patients with colitis	Used in conjunction with gemcitabine and nab-paclitaxel
			NCT01560923	II	Prostate cancer	Median PFS increased from 4.1 to 10.3 months	No significant AEs	Ongoing
		Epacadostat	NCT02327078	I/II	Melanoma, NSCLC, CRC, HNSCC, glioblastoma, ovarian cancer, and HD	ORR 75% and DCR 100% in melanoma, ORR 11% and DCR 28% in ovarian cancer, ORR 4%, and DCR 24% in CRC	No dose-limiting toxicities	In conjunction with nivolumab
			NCT02178722	I/II	Solid tumors and NHL	–	37/244 patients with grade 3 or more AEs, 3% discontinued therapy due to AEs	In conjunction with pembrolizumab
			NCT01195311	I	Solid tumors	No OR, 7/25 patients achieved SD	1/52 patients developed grade 3 pneumonitis, 1/52 developed grade 3 fatigue	Completed
	TLR	MEDI9197	NCT02556463	I	Solid tumors	–	No severe AEs	In combination with durvalumab and radiation therapy
		PG545 (pixatimod, pINN)	NCT02042781	I	Solid tumors	DCR 38%	3/23 patients developed dose-limiting toxicities	Completed
		Polyinosinic-polycytidylic acid polylysine carboxymethylcellulose (poly-ICLC)	NCT00553683	I	HCC	PFS 66% at 6 months and 28% at 24 months, OS 69% after 1 year and 38% after 2 years	1/18 patients with severe AE with hepatic artery embolization	Completed
	IL-2R	NKTR-214	NCT02983045	I/II	Solid tumors	1 patient with melanoma had a mixed radiographic response, another melanoma patient had an unconfirmed CR	No dose-limiting toxicities	In conjunction with nivolumab. Ongoing
	Arginase inhibitors	CB-1158	NCT02903914	I	Solid tumors	–	No dose-limiting toxicities	Alone and in conjunction with nivolumab
	Oncolytic peptides	LTX-315	NCT01986426	I	Melanoma and BC	2/28 patients CR, 5/28 showed > 50% decrease in tumor size, 8/28 SD	Grade 1 and 2 EAs	Monotherapy or in conjunction with ipilimumab or pembrolizumab
	IL-10	AM0010	NCT02009449	I	Melanoma	DCR 45%	11/25 patients with grade 3–4 AEs	In conjunction with pembrolizumab

*Abbreviations*: *AE* adverse event, *BC* breast cancer, *CNS* central nervous system, *CR* complete response, *CRC* colorectal cancer, *CTCL* cutaneous T cell lymphomas, *DART* dual affinity re-targeting, *DCR* disease control rate, *HCC* hepatocellular carcinoma, *HD* Hodgkin’s disease, *HNSCC* head and neck squamous cell carcinoma, *IDO* indoleamine 2,3-dioxygenase, *NHL* non-Hogkin’s lymphoma, *NSCLC* non-small cell lung carcinoma, *OS* overall survival, *OR* objective response, *ORR* objective response rate, *PD* progressive disease, *PFS* progression-free survival, *PR* partial response, *RCC* renal cell carcinoma, *SCLC* small cell lung cancer, *SD* stable disease, *TLR* toll-like receptor

## Inhibitory pathways

Binding of CTLA-4 and PD-1/PD-L1 to cancer cell or tumor-microenvironmental ligands leads to T cell attenuation, which enables the tumor cells to avoid immune-mediated destruction [1]. Similarly, other inhibitory pathways have been identified and new blocking agents are being developed to induce immune reaction against malignant cells [4]. These inhibitory pathways can be classified as T cell associated and non-T cell associated, as follows.

### T cell-associated inhibitory molecules

#### LAG-3 (CD223)

Lymphocyte activation gene-3 (LAG-3, CD223) is expressed by T cells and natural killer (NK) cells after major histocompatibility complex (MHC) class II ligation [9, 10]. Although its mechanism remains unclear, its modulation causes a negative regulatory effect over T cell function, preventing tissue damage and autoimmunity. LAG-3 and PD-1 are frequently co-expressed and upregulated on tumor-infiltrating lymphocytes (TILs) leading to immune exhaustion and tumor growth [11]. Thus, LAG-3 blockade not only improves anti-tumor immune responses but also potentiates other forms of immunotherapy given its different mechanism of action mainly mediated by impeding cell cycle progression [12–14]. Although simultaneous use with anti-PD-1 therapy is considered synergistic, it remains unclear whether other immune checkpoint inhibitory molecules in conjunction with anti-LAG-3 therapy will be as effective [15]. Furthermore, clinical benefits from combination come at the expense of increased incidence of autoimmune toxicities [1]. Currently two inhibitory approaches have been developed: a LAG-3-Ig fusion protein (IMP321, Immuntep®) and mAbs targeting LAG-3 [5].

IMP321, a soluble form of LAG-3, upregulates co-stimulatory molecules and increases interleukin (IL)-12 production to enhance tumor immune responses. Two phase I clinical trials using IMP321 in advanced renal cell carcinoma (RCC) and pancreatic adenocarcinoma showed an increase in tumor reactive T cells, but no meaningful objective response (OR) was observed [16, 17]. Another phase I clinical trial studied IMP321 in combination with paclitaxel in metastatic breast cancer (BC) and an objective response rate (ORR) of 50% was observed [18]. This promising result has prompted a phase IIb clinical trial that is currently recruiting patients with metastatic BC (NCT02614833).

Targeting LAG-3 with antagonistic mAbs interferes with the LAG-3 interaction between MCH II molecules expressed by tumor and/or immune cells, promoting tumor cell apoptosis [19]. A phase I clinical trial is recruiting melanoma patients to determine the safety of anti-LAG-3 (BMS-986016), with and without nivolumab (NCT01968109). Interim results show promising efficacy with an ORR of 16% and disease control rate (DCR) of 45% among patients who had progressed despite previous therapy with anti-PD-1/PD-L1. The safety profile is similar to nivolumab alone [20]. LAG525 is another anti-LAG-3 mAb being studied on a phase I/II clinical trial with metastatic solid malignancies (NCT02460224), and currently no data is available.

#### TIM-3

T cell immunoglobulin-3 (TIM-3) is a direct negative regulator of T cells and is expressed on NK cells and macrophages. TIM-3 indirectly promotes immunosuppression by inducing expansion of myeloid-derived suppressor cells (MDSCs). Its levels have been found to be particularly elevated on dysfunctional and exhausted T cells suggesting an important role in malignancy [21]. Presence of TIM-3+ T cells correlates with severity and poor prognosis in non-small cell lung carcinoma (NSCLC) and follicular lymphoma [11]. On the other hand, low levels of TIM-3 have been associated with autoimmune processes such as in diabetes or multiple sclerosis [22]. Similarly, the use of monoclonal antibodies to block TIM-3 causes an increase in T cell proliferation and cytokine production which may not only explain its antitumor activity but also its role in aggravating autoimmune diseases [22]. Furthermore, there has been concern with the use of these antibodies given that TIM-3 could act as an enhancer of CD8 T cells during certain acute infections including Listeria [23].

Modulation of this pathway occurs through multiple ligands including galectin-9, phosphatidyl serine, and CEACAM-1 [11]. These molecules play an important role in carcinogenesis, tumor survival, and even progression of different malignancies including melanoma, gastrointestinal, and lung cancer [24–26]. As opposed to other inhibitory pathways that interfere with cellular function, TIM-3 primarily exert its function by regulating cell apoptosis [27]. This could potentially explain its enhancing effects when used with other ICIs. However, the best complementary molecule to be used with TIM-3 remains unknown.

Currently, one anti-TIM-3 mAb (MBG453) is being investigated in phase I–II clinical trial in patients with advanced malignancies (NCT02608268). No clinical results are yet reported.

#### TIGIT

T cell immunoglobulin and ITIM domain (TIGIT) is part of the CD28 family-like receptors expressed by NK and T cells. It exerts direct immunosuppressive effects on these cells and indirectly increases the release of immunoregulatory cytokines (e.g., IL-10), decreases the production of interferon (IFN)-γ and IL-17, and prevents maturation of DCs [28, 29]. Two agonists, CD155 (poliovirus receptor-PVR) and CD112 (PVRL2, nectin-2), are expressed by immune cells, non-immune cells, and by tumor cells including melanoma [30]. Moreover, TILs often express high levels of TIGIT along with PD-1, TIM-3, and LAG-3, consistent with a dysfunctional phenotype [31].

Initial ex vivo and murine studies targeting dual blockade of TIGIT and either PD-1 or TIM-3 has shown a synergistic effect in immune cell proliferation, cytokine release, degranulation, and reversal of T cell exhaustion with subsequent tumor rejection and induction of protective memory responses [11, 32]. Importantly, the expression of TIGIT appears to be higher in the cells within tumor microenvironment than in those in the periphery, which would theoretically offer the advantage of a more targeted-directed therapy with less systemic autoimmune-like toxicities. Furthermore, TIGIT appears to exert its effects primarily by limiting cytokine competency and CD8 T cell function which would in theory explain its complementary effects when used with other forms of ICIs [27].

A phase I clinical trial is currently recruiting patients to evaluate the safety and efficacy of the anti-TIGIT mAb OMP-31M32 (NCT03119428). No results are yet available.

#### VISTA

V-domain Ig suppressor of T cell activation (VISTA), also known as programmed death-1 homolog (PD-1H), is a unique molecule with dual activity. It behaves as a stimulatory ligand for antigen presenting cells (APCs) causing immune activation and as a negative ligand for T cells suppressing activation, proliferation, and cytokine production [33]. Le Mercier et al. demonstrated that its blockade improved TIL activation and enhanced tumor-specific T cell responses in the periphery despite the presence of high PD-L1 levels or the lack of expression of VISTA within tumor cells [34]. Therefore, both pathways are considered independent and simultaneous dual blockade of PD-1 and VISTA is often viewed as synergistic [35]. Interestingly, VISTA expression levels appear to vary among different tumors, often seen as a limitation given theoretical response heterogeneity. However, its blockade has proven to be effective even in the absence of detectable levels which offers the advantage of a broader clinical applicability but poses the challenge of finding specific biomarkers to predict response [35]. Additionally, this pathway is expressed mainly by TILs which, similar to TIGIT, allow it to be more tumor-specific and less toxic than other pathways.

Two molecules are being tested on phase I clinical trials: JNJ-61610588, a fully human mAb against VISTA, and CA-170, an oral inhibitor of both PD-L1/PD-L2 and VISTA. Both trials are currently recruiting (NCT02671955, NCT02812875).

#### B7-H3 (CD276)

B7 homolog 3 (B7-H3), also known as CD276, is a protein that belongs to the B7-CD28 pathway family and is widely expressed in different solid organs as well as immune cells including APCs, NKs, and B and T cells. It has an inhibitory function on T cell activation, proliferation, and cytokine production [36]. Furthermore, this pathway appears to promote cancer aggressiveness. Thus, blocking this agent would not only offer the advantage of enhancing innate immunological responses against malignancy but also would exert a direct effect over tumor behavior. B7-H3 expression is limited on healthy tissues but overexpression is common in multiple malignancies including melanoma, NSCLC, prostate, pancreatic, ovarian, and colorectal cancer (CRC) [36, 37]. Therefore, developing strategies to block this pathway would offer the advantage of exerting more localized effects over malignancies with less prominent systemic toxicities. Additionally, given its unique mechanism of action compared to other anticancer strategies, B7-H3 appears to have a synergistic effect when combined with chemotherapy or other ICIs [36].

Enoblituzumab (MGA271) is an engineered Fc humanized IgG1 monoclonal antibody against B7-H3 with potent anti-tumor activity. Interim results of an ongoing phase I clinical trial using MGA271 in melanoma, prostate cancer, and other solid tumors (NCT01391143) shows that it is overall well-tolerated without dose-limiting toxicities. Disease stabilization and objective responses ranging from 2 to 69% were noted across several tumor types [38]. Another phase I clinical trial is evaluating the use of enoblituzumab in combination with pembrolizumab (NCT02475213). Both studies are currently recruiting.

The use of dual affinity re-targeting (DART) proteins that bind both CD3 on T cells and B7-H3 on the target cell has been found to recruit T cells to the tumor site and promote tumor eradication [39]. MGD009 is a humanized DART protein that is being studied on a phase I clinical study in patients with B7-H3 expressing tumors including melanoma, NSCLC, mesothelioma, and urothelial cancers [40]. The trial is ongoing and recruiting patients (NCT02628535).

8H9 is an antibody against B7-H3 labeled with radioactive iodine (I-131) which, after internalization, promotes cancer cell death [36]. This drug has been tested on metastatic neuroblastoma in conjunction with radiation therapy and surgery [41]. The ongoing trial is assigning patients to be treated with either mAbs against B7-H3 or against GD-2 (NCT00089245). Preliminary results revealed that 17/21 patients studied were alive and free of disease after a median follow-up of 33 months [41]. 8H9 is also being studied on peritoneal cancers, gliomas, and advanced central nervous system malignancies (NCT01099644, NCT01502917, NCT00089245).

#### A2aR and CD73

The adenosine pathway encompasses specific adenosine receptors and enzymes that synthetize it. Adenosine A2a receptor (A2aR) is one of the most important factors in this pathway and is mainly activated by adenosine [1]. A2aR is expressed on immune cells, including T cells, APCs, NK cells, and on endothelial cells. Increased levels of adenosine in tumor microenvironment can promote formation of Treg cells and can dampen the immune response of multiple effectors including macrophages, NK, APCs, and neutrophils [42]. CD73, on the other hand, is widely expressed by most tissues and is thought to serve as an adhesion molecule for lymphocyte binding to the endothelium and to play an important role as a co-signal for T lymphocyte activation. However, it also widely expressed by malignant cells where it acts as an enzyme and promotes the formation of adenosine by the dephosphorylation of AMP, favoring tumor progression [43]. Not surprisingly, often these molecules are overexpressed in various malignancies and usually correlate with poor overall prognosis [44]. Given the multiple mechanisms that interact in this pathway and its importance in tumor microenvironment, different strategies to target both A2aR and CD73 have been developed. The main advantage of this approach is the potential use of combination strategies with other forms of therapy including chemotherapy or other ICIs. Furthermore, the use of combination strategies among the adenosine pathway is an additional possibility [44]. However, an area of concern with this approach is the blockade of adenosine synthetic enzymes which may favor the accumulation of ATP, a molecule that can play a pro-tumor role in the tumor microenvironment [44]. An additional limitation, as with other forms of immunotherapy, is the lack of clinical or biological markers that help with stratification of patients that are most likely to benefit from this form of therapy.

Blockade of A2aR on mice demonstrated increased proliferative capacity and function of T cells, as well as enhanced immunologic memory [42]. Preliminary results from a phase I clinical trial evaluating the oral adenosine A2aR antagonist CPI-444 alone and in combination with atezolizumab for advanced solid cancer showed that 42% of patients (10 of 24) who had been resistant to anti-PD-1/PD-L1 therapy, achieved disease control. Furthermore, grade 1 and 2 toxicities were the most common with only one case of grade 3 autoimmune hemolytic anemia [45]. This trial is ongoing and recruiting patients (NCT02655822).

MEDI9447 is a monoclonal antibody specific for CD73 that is being studied on a first-in-human clinical trial in patients with advanced solid tumors that have progressed or are refractory to standard therapy (NCT02503774). No preliminary results are yet available. Of note, CD73 could play a role in tumor angiogenesis; however, no studies have been designed yet to evaluate a possible synergistic effect of anti-CD73 and antiangiogenic therapy [46].

#### BTLA

B and T cell lymphocyte attenuator (BTLA, CD272) is an inhibitory receptor that is structurally and functionally related to CTLA-4 and PD-1 and is expressed by the majority of lymphocytes. Ligation of BTLA by its ligand, herpes virus entry mediator (HVEM), blocks B and T cell activation, proliferation, and cytokine production [47]. Tumor cells exploit this pathway by either promoting the formation of dysfunctional T cells that persistently express BTLA and render them susceptible to inactivation, or by expressing HVEM, as it has been found with melanoma [47]. High levels of BTLA/HVEM on melanoma and gastric cancer patients correlate with poor prognosis [48, 49]. Thus, the BTLA-HVEM pathway is being considered as a new target for checkpoint blockade [48]. The main limitation with this form of therapy has been the complexity of the receptor-ligand system. Additionally, given a different mechanism of action compared to other forms of immunotherapy, combination with other molecules could be synergistic but also be associated with an increased risk of toxicity [47].

### Non-T cell-associated inhibitory molecules

#### TGF-β

Transforming growth factor (TGF)-β is a cytokine that helps maintain tissue homeostasis by regulating cellular growth, differentiation, proliferation, and survival [50]. Although this pathway is able to control early-stage tumors by promoting cell cycle arrest and apoptosis, in advanced stages, it allows for tumor evasion by suppressing cytotoxic T cells and promotes cancer cell proliferation, invasion, and metastases, a functional switch known as the “TGF-β paradox” [51, 52]. Malignant cells achieve this switch through either the inactivation of their TGF-β receptors, or by selectively disabling the tumor-suppressive arm of this pathway, allowing cancer cells to use the TGF-β regulatory functions to their advantage by promoting immune tolerance [53]. In fact, tumors that produce high levels of TGF-β can shield themselves from immune surveillance [50]. Consistently, increased TGF-β expression by NSCLC, CRC, gastric, and prostate cancer has correlated with tumor progression and poor prognosis [50].

Many malignant cells have an abnormal TGF-β signaling pathway and blocking agents exert an indirect action mainly by acting over the cells within the tumor microenvironment [54]. This allows for potential combination with other forms of therapy including immune checkpoint targeting and chemotherapy. Some challenges to note with this approach include the lack of biomarkers that allow defining the microenvironment where these agents are most usefuland the potential risk of synchronous occult tumor growth by inhibiting the TGF-β suppressive action in early-stage cancers [54]. There are three methods for blocking the TGF-β pathway: blocking the ligand, ligand-receptor interaction, or the receptor tyrosine kinase activity. Trabedersen [AP12009], a synthetic antisense oligonucleotide that hybridizes with RNA sequences and blocks TGF-β translation, has been tested on patients with glioblastoma multiforme and anaplastic astrocytoma [55, 56]. It was also tested on advanced pancreatic cancer where OS improved by 9.9–11.8 months although no improvement on progression-free survival (PFS) was observed [57].

M7824 is a dual anti-PD-L1 monoclonal antibody fused with a soluble extracellular domain of TGF-β receptor II, which acts as a TGF-β trap. A phase I clinical trial is being conducted on patients with metastatic or locally advanced solid tumors using this novel chimeric molecule (NCT02517398). Preliminary results from a trial in 16 patients demonstrate an acceptable safety profile with no grade 4–5 adverse events. Preliminary assessments suggest clinical benefit with one patient demonstrating a CR, one with durable PR, one patient with a 25% reduction of target lesions after two doses, and two cases with prolonged stable disease (SD) [58].

Galusertinib (LY2157299), a blocker of the receptor’s tyrosine kinase activity was tested in a recent phase II clinical study, but failed to demonstrate improved OS compared to placebo [59]. This molecule is being studied on NSCLC, hepatocellular carcinoma (HCC), pancreatic cancer, and BC (NCT02423343, NCT02734160, and NCT02672475).

#### KIR

Killer immunoglobulin-like receptors (KIRs, CD158) are a family of transmembrane proteins that promote self-tolerance by dampening lymphocyte activation, cytotoxic activity, and cytokine release. They are expressed by NK cells and some T cells and assist with self-recognition of host cells through the binding of MHC-I. KIR aids in the identification and destruction of cells that have lost their MHC-I as with many tumor cells, a process termed “missing self” recognition [60]. Some malignancies, however, develop mechanisms to evade this pathway by either upregulating non-classical MHC-I molecules or by changing the tumor microenvironment properties rendering NK cells dysfunctional [61].

The use of monoclonal antibodies to manipulate the KIR pathway is an active area of investigation as interfering with MHC-I interactions can stimulate NK cells by mimicking the “missing self” response [62]. The main advantage of targeting KIR is activating mostly NK rather than T cells, which is a potentially synergistic antitumor approach by allowing T cell ligands be available for targeting with other forms of immunotherapy. However, given its importance in self-recognition, NK cell overactivation may lead to a proinflammatory state and increase the risk for autoimmune reactions [63]. Different molecules targeting KIR are under investigation. Lirilumab, a fully human monoclonal antibody that blocks KIR2DL1/2L3, is currently being studied in a phase I/II clinical trial with concurrent use of nivolumab and ipilimumab in patients with squamous cell carcinoma of the head and neck (NCT01714739). Preliminary results are promising, with an ORR of 24% and a DCR of 52%, and only 8% of patients stopping therapy due to adverse events [64].

KIR3DL2 is frequently expressed by cutaneous T cell lymphomas (CTCL) and has prognostic and diagnostic features within this population [65]. IPH4102 is a monoclonal antibody against kIR3DL2 which is currently being investigated in a phase I clinical trial in patients with relapsed or advanced CTCL (NCT02593045). Preliminary results reveal an ORR of 45%; 10 out of 22 patients with PR, 2 CR in skin, and 5 CR in blood. Six patients developed grade 3 or more severe adverse events [66].

#### PI3Kγ

The expression of Phosphoinositide 3-kinase gamma (PI3Kγ) by macrophages controls a critical switch towards immune suppression in presence of inflammation and cancer. Additionally, PI3Kγ seems to play a role in angiogenesis by affecting the function of tumor-associated macrophages, major producers of VEGF [67]. Thus, similar to TGF-β, blocking this pathway exerts an indirect antitumor effect by modifying the microenvironment, improving the immunological function against malignant cells, and affecting the tumor vasculature. Unfortunately, as with other forms of immunotherapy, blocking PI3K enzymes has been associated with multiple autoimmune-like toxicities, and therefore the use of lower doses in conjunction with other forms of immunotherapy is often used [67].

IPI-549 is an oral selective inhibitor of PI3Kγ being studied on a phase I clinical trial as monotherapy or in combination with nivolumab in patients with melanoma, NSCLC, or head and neck cancer (NCT02637531). Preliminary results demonstrate no dose-limiting toxicities and only mild adverse events including nausea and fatigue. Importantly, 12 out of 15 patients have demonstrated durable clinical benefit, and 50% of patients have been able to remain on treatment ≥ 16 weeks [68].

#### CD47

CD47, also known as integrin-associated protein, is a molecule that exerts its action through the signal regulatory protein alpha (SIRPα). It is ubiquitously expressed by healthy cells to help with autologous recognition and avoid inappropriate phagocytosis [69]. Solid tumors (e.g., bladder and BC) and hematologic cancers (e.g., acute myeloid leukemia and non-Hodgkin’s lymphoma) overexpress CD47 causing an inhibitory effect over macrophages and other myeloid cells and high levels of CD47 correlate with poor prognosis [69]. The blockade of the CD47/ SIRPα axis results in an increased macrophage recruitment and antitumor activity through phagocytosis and cytokines secretion. However, the use of this pathway has pointed certain limitations mainly derived from the diffuse expression of CD47. First, a potential “antigen sink” effect where high doses may be required to achieve an appropriate therapeutic blockade [70]. Second, there is an increased risk for “on-target” systemic toxicities over healthy cells that express CD47. Until now, therapy has been overall well tolerated and anemia has been the most common adverse event [70]. Hu5F9-G4, a humanized monoclonal antibody targeting CD47, is being studied on a phase 1 clinical trial in patients with solid tumors (NCT02216409). In preliminary results, it showed acceptable tolerability and SD in 2 out of 16 patients for 16 and 8 months, respectively [71]. Another phase I/II clinical trial using this molecule in combination with rituximab in patients with relapsed or refractory B cell non-Hodgkin’s lymphoma is still recruiting patients (NCT02953509).

TTI-621 (SIRPαFc) is a fully recombinant fusion protein consisting of a CD47 binding domain linked to the Fc region of IgG1 to block the CD47 “do not eat me” signal and engage macrophage Fcγ receptors to enhance phagocytosis and antitumor activity [72]. A phase I clinical trial using TTI-621 in patients with relapsed or refractory percutaneously accessible solid tumors and mycosis fungoides is currently recruiting patients (NCT02890368).

## Co-stimulatory pathways

As opposed to inhibitory pathways that attenuate the immune system, co-stimulatory molecules augment immunological responses against malignant cells. Malignant cells inhibit these pathways to promote tumorigenesis [5].

### OX40

OX40 (CD134) is a member of the TNF receptor super family, highly expressed by activated CD4, CD8 T cells, and Tregs, and in a lesser degree by neutrophils and NK cells. This molecule, along with its ligand, OX40L, plays a pivotal role in activation, potentiation, proliferation, and survival of T cells and modulation of NK cell function [73]. Furthermore, this molecule inhibits the suppressive activity of Tregs by directly interfering with their function and proliferation, and indirectly antagonizing their inhibitory byproducts (e.g., TGFβ) [74]. Importantly, when tumor antigens are recognized by TILs, its expression of OX40 increases, and not surprisingly, the amount of OX40-expressing TILs correlates with improved prognosis in certain populations [75].

The use of mAbs to activate OX40 has been a strategy used to increase the antitumor activity by the immune system. Of note, these antibodies have been associated with depletion of TILs through an antibody-dependent cell cytotoxicity. NK cells recognize the antibodies bound to antigens over cell surfaces and kill these cells [76]. However, this only occurs in the presence of NKs within the tumor, which varies depending on the host and type of malignancy. Another limitation is a potential activation of peripheral lymphocytes rather than TILs when therapy is given systemically. Thus, its intratumoral administration has been proposed as a way to minimize systemic toxicity [76]. Despite its limitations, use of these antibodies has demonstrated tumor regression in several preclinical models, although often are used in conjunction with other forms of immunotherapy [75]. 9B12 is a murine IgG monoclonal agonistic antibody against OX40 that was studied in a phase I clinical trial in 30 patients with metastatic solid malignancies [77]. Although no patients achieved PR, SD was achieved in 6 patients. Adverse events were overall tolerable and limited to grades 1 and 2 except for transient lymphopenia which was found to be grade 3 or more in 7 patients [77].

MOXR 0916 is a humanized IgG agonistic monoclonal OX40-specific antibody that is currently being tested in combination with atezolizumab in patients with advanced solid malignancies (NCT02410512). Preliminary results show no dose-limiting toxicities but efficacy results are not yet available [78]. PF-04518600 (PF-8600) is an IgG2 humanized agonistic monoclonal antibody of OX40 that is undergoing a first-in-human trial (NCT02315066). Preliminary results in patients with selected advances solid tumors including melanoma and NSCLC revealed no dose-limiting toxicities, and 4 out of 9 patients demonstrated SD [79].

MEDI6383, MEDI0562, MEDI6469, INCAGN01949, and GSK3174998 are other agonistic monoclonal antibodies that are part of different phase I clinical trials for which no preliminary results are yet available (NCT02221960, NCT02528357, NCT02923349, NCT02705482).

### GITR

Glucocorticoid-induced TNF receptor family-related protein (GITR) is a co-stimulatory cell surface receptor that is constitutively expressed by T cells and NK cells, and expression increases markedly following T cell activation. Its ligand, GITRL, is mainly expressed by APCs and endothelial cells and appears to have a role in upregulating the immune system, leukocyte adhesion, and migration [80]. The expression of GITR by TILs in the tumor microenvironment has been found to be higher than levels expressed by peripheral lymphocytes, indicating local T cell activation [80]. Agonizing agents of this pathway have been considered as a way to increase the immune antitumor activity, although the clinical utility of such agents depends on the presence of T cells in the tumor and the subset of TILs which may vary among different malignancy [81]. Thus, selection of patients who will derive the most benefit from this therapy is still unclear. Immune-related adverse events should also be considered. Preclinical data suggests that GITR therapy appears to be better tolerated than anti-CTLA4 agents [81].

GITR modulation in the preclinical models has shown promising antitumor activity via significant increase in effector T cells and decrease in Tregs [80]. TRX-518, an aglycosylated human mAb that agonizes GITR, is currently undergoing phase I clinical study in various solid malignancies (NCT01239134). Preliminary results demonstrate an acceptable safety profile without dose-limiting toxicities and SD in 10% of study patients (4 out of 40 patients) [82]. BMS-986156 is another anti-GITR antibody that being studied in a phase I clinical trial alone or in combination with nivolumab in patients with advanced solid tumors (NCT02598960). Preliminary results showed no dose-limiting toxicities, though no efficacy results were reported [83]. AMG 228, an agonistic IgG1 monoclonal antibody of GITR, was also recently studied in a first-in-human clinical trial in 30 patients with refractory CRC, head and neck squamous cell carcinoma, urothelial carcinoma, and melanoma [84]. None of the patients demonstrated OR, and no dose-limiting toxicities were identified. Up to 90% of patients (27/30) experienced adverse events consisting of electrolyte imbalances, anemia, and fever [84].

Other similarly agents including MEDI1873, MK-4166, INCAGN01876, and GWN323 are also being studied in multiple solid and hematologic malignancies (NCT02583165, NCT02132754, NCT02697591, NCT03126110, NCT02740270).

### ICOS

Inducible co-stimulator (ICOS), a specific T cell co-stimulatory molecule of the CD28/CTLA-4 family mainly expressed by CD4 T cells, is a co-stimulator of proliferation and cytokine production by these cells [85]. Its levels are upregulated in activated T lymphocytes, especially after the use of anti-CTLA4 therapies, and its expression is considered a biomarker to indicate that anti-CTLA4 agents are binding its target [86]. Increased ICOS expression on circulating T cells after ipilimumab administration has been associated with improved clinical outcomes [87]. Interestingly, ICOS appears to be a less potent pathway compared to other forms of immunotherapy mainly because of a predominant CD4 expression. However, its use with other approaches, particularly CTLA4 blockade, can lead to a potent synergistic effect as a result of an increase in the expression of ICOS after anti-CTLA4 therapy [85].

Some molecules have been developed and are being investigated. JTX-2011 is an agonistic monoclonal antibody of ICOS that is currently being tested in a phase I/II clinical trial alone and in combination with nivolumab in patients with advanced solid malignancies including endometrial, breast, lung, pancreatic, and CRC (ICONIC Trial—NCT02904226). Preliminary results showed no dose-limiting toxicities, although efficacy is not reported [88]. Similarly, GSK3359609 is a humanized IgG4 monoclonal agonistic antibody of ICOS that is undergoing clinical investigation in a phase I clinical trial, alone or in combination with pembrolizumab in patients with advanced solid tumors (INDUCE-1 trial - NCT02723955). Finally, MEDI-570, an agonist monoclonal IgG1 antibody directed against ICOS is also being studied in a phase I clinical trial in patients with Non-Hodgkin lymphomas (NCT02520791).

### 4-1BB

4-1BB (CD137) is an inducible co-stimulatory receptor expressed by T cells, NK cells, and APCs. Once expressed, it binds its ligand (4-1BBL) and triggers subsequent immune cell proliferation and activation, particularly of T and NK cells [89]. The activation of NK cells leads to an increased antibody-dependent cell-mediated toxicity. Thus, the use of anti-41BB agonists not only increases immune-mediated antitumor activity but is also considered an ideal agent to use in combination with other monoclonal antibodies such as rituximab and trastuzumab [89]. Of note, the use of 4-1BB antibodies in conjunction with other ICIs may lead to an important antitumor response with potential increased toxicity. In fact, given the diffuse expression of 4-1BB, there is a notorious risk for “on-target” systemic adverse events [89].

These antibodies have been expanded to clinical studies after demonstrating potent anti-cancer efficacy in murine models [90]. Utomilumab (PF-05082566), a fully human mAb that stimulates 4-1BB, has been studied in a phase I clinical trial in combination with pembrolizumab in patients with advanced solid tumors [91]. No dose-limiting toxicities were reported and 6 out of 23 patients had either CR or PR. This drug is currently being studied in multiple phase I clinical trials: alone or in various combinations with rituximab (NCT01307267), mogamulizumab (NCT02444793), an experimental OX40 agonist (NCT02315066), and avelumab (NCT02554812).

Urelumab is another agonist antibody of 4-1BB that has been studied in various clinical trials in patients with advanced solid tumors. A safety analysis from these trials concluded that this agent can occasionally cause significant transaminitis when high doses are used [92]. Currently, this medication is being evaluated in combination with nivolumab in a phase I/II clinical trial in patients with solid tumors and B cell non-Hodgkin’s lymphoma (NCT02253992). Preliminary results showed that 6/60 of the patients with lymphoma treated with urelumab monotherapy achieved a PR (*n* = 3) or CR (*n* = 3), 9/86 patients who received combination therapy achieved PR although none of the patients with NSCLC or diffuse large B cell lymphoma had reported response. Of note, at least 3% of patients developed grade 3–4 transaminitis, and 7% of the 123 enrolled patients developed serious adverse events leading to discontinuation in 5% of study patients [93]. Another phase I clinical trial evaluating urelumab in combination with rituximab is being conducted in patients with metastatic solid tumors and refractory NHL (NCT01471210). No results have been yet published.

According to a recent comparison between urelumab and utomilumab, the former seems to exert a more marked agonistic activity on the receptor [94].

### CD27-CD70

Binding of CD27, a member of the TNF receptor family, with its ligand CD70, results in a potent signal to activate and differentiate T cells into effector and memory cells, and to boost B cells [95]. Despite its wide spectrum of action, this pathway has not demonstrated to be particularly effective in overcoming the immunosuppressive features of the tumor microenvironment. Thus, CD27 is considered most useful as combination rather than monotherapy. Furthermore, its use with other blocking agents like anti-CTLA-4 or anti-PD-1/PD-L1 may not only be synergistic but also associated with less autoimmune toxicities [96]. When used as monotherapy, CD27 agonist has been well tolerated and only minor adverse events are reported. An important aspect in this pathway is the identification of CD27 phenotype on tumor, as cancers that express this molecule could achieve a more favorable outcome [96].

The use of CD27-CD70 agonist agents has been evaluated in various preclinical settings and is being studied in multiple clinical trials. ARGX-110 is an agonistic anti-CD70 monoclonal antibody that has been studied in a phase I clinical trial in patients with T cell lymphoma [97]. Of note, 2 out of 9 patients had a reduction of malignant clones of > 90%, one patient achieved radiological PR, and 2 patients reached PR in the skin. Currently one phase I clinical trial is recruiting patients with advanced malignancies (NCT01813539). BMS-936561 (MDX-1203) is another fully human monoclonal agonistic CD70-specific antibody that was studied in RCC and B cell lymphoma [98]. Results demonstrated disease stabilization in 69% of treated individuals. Varlilumab, a monoclonal agonistic antibody against CD27, is currently under investigation in a phase I clinical trial with simultaneous use of nivolumab in patients with advanced solid tumors (NCT02335918). Preliminary results showed a notable increase of TILs in post-treatment biopsies [99]. Currently this molecule is being studied in other phase I and II clinical trials in patients with gliomas, melanomas, RCC, and other solid tumors (NCT02924038, NCT02302339, NCT02386111, NCT02543645).

### CD40

CD40 is a member of the TNF receptor family expressed by APCs and B cells whereas its ligand, CD154, is expressed by activated T cells. Interaction between CD40-CD154 stimulates cytokines secretion of B cells with subsequent T cell activation and tumor cell death [100]. Despite its potential synergy with other forms of anticancer therapy, the use of CD40 agonists has also been associated with particular toxicities including cytokine release syndrome, thromboembolic events, and tumor angiogenesis. It is probably related to the expression of CD40 by platelets and endothelial cells [101]. The main challenges that remain with this particular form of therapy include the identification of appropriate combinations and patient population that would benefit from these agents. As of now, eight mAbs have entered clinical trials: CP-870893, APX005M, ADC-1013, lucatumumab, Chi Lob 7/4, dacetuzumab, SEA-CD40, and RO7009789. Some of these were recently reviewed [102, 103]. Others are still under investigation (NCT02482168, NCT03165994, NCT02706353, NCT03123783, NCT02829099, NCT02588443, NCT02760797, NCT02665416, NCT02304393).

## Other potential pathways

### IDO

Indoleamine 2,3-dioxygenase (IDO) is a tryptophan-degrading enzyme that converts tryptophan to kynurenines. Kynurenines promote the differentiation and activity of Treg and decrease the amount and activity of CD8 T cells leading to an immunosuppressed environment only worsened by the high levels of PD-1/PD-L1 concurrently present in this milieu [104]. IDO has been found overexpressed in various tumor cell types including melanoma, chronic lymphocytic leukemia, ovarian, CRC, and more recently in sarcomas [104, 105]. Furthermore, high levels of IDO not only correlate with poor outcomes in some malignancies but may also be involved in drug resistance to chemotherapeutic agents [106]. Though their ability to counterbalance the immunosuppressive tumor microenvironment is promising, treatment with IDO inhibitors has also raised specific concerns. First, IDO is induced by inflammatory molecules such as IFNγ. Therefore, the lack of inflammation in the tumor microenvironment may be associated with a suboptimal response to anti-IDO agents [106]. Second, IDO and other similar enzymes are also expressed by healthy tissue, and its inhibition may lead to cross-reaction side effects. Regardless, IDO inhibitors remain a great area of interest among immune checkpoint therapy and different molecules are under investigation.

BMS-986205 is a once-daily, selective, and potent oral IDO1 inhibitor that is currently undergoing in a phase I clinical trial with concomitant use of nivolumab (NCT02658890). All reported toxicities have been grades 1–2 except for three cases of grade 3 hepatitis, rash, and hypophosphatemia. No efficacy was reported [107].

Indoximod is another IDO inhibitor that is being studied in phase II clinical trials in melanoma (NCT02073123), pancreatic cancer (NCT02077881), and castrate-resistant prostate cancer (CRPC) (NCT01560923). Results seem promising. ORR was 52% in patients with melanoma in whom indoximod was given with either ipilimumab, nivolumab, or pembrolizumab [108]. Patients with pancreatic cancer had an ORR of 37% with concomitant use of indoximod, gemcitabine, and nab-paclitaxel [109]. With indoximod, median PFS has increased from 4.1 to 10.3 months in metastatic CRPC compared to placebo [110].

Finally, epacadostat is another oral agent that blocks IDO pathway and is undergoing investigation in phase I/II clinical trials evaluating multiple malignancies (NCT02327078, NCT02178722). Preliminary results have demonstrated an ORR ranging from 75% in melanoma to 4% in CRC. Its use seems to be safe with pembrolizumab. Although no dose-limiting toxicities have been identified, up to 3% of patients have discontinued therapy due to adverse events [111, 112]. In another completed phase I clinical trial with 52 patients who had advanced solid tumors (INCB024360), treatment with epacadostat demonstrated overall well tolerable adverse reactions except for 1/52 grade 3 pneumonitis and 1/52 grade 3 fatigue. No OR was reported, but 7/52 patients achieved SD greater than 16 weeks [113].

### TLR

Toll-like receptors (TLRs) are considered critical in the recognition of pathogens and control of the immune response. However, their role in tumorigenesis is far more complex. Some TLRs, like TLR4, may promote cancer progression by either favoring inflammation in the tumor microenvironment or inducing Tregs or PD-L1. Other TLRs like TLR7/8 and TLR9, induce antitumor responses by promoting a “danger signal” within the tumor microenvironment and activating the immune system against malignant cells [114]. The use of agents to manipulate these TLRs pathways seem to not only promote an immune response against malignancy but also induce autophagy and apoptosis of cancer cells [115]. There are certain important aspects to note with TLR therapy. First, its non-specific capability of inducing not only cytotoxic T cells but also immunosuppressive cells within the tumor microenvironment leads to an overall attenuated tumoricidal effect [116]. Second, an appropriate combination partner and identification of patients that would benefit the most of these agents remains unclear. It has been established that concomitant use of these molecules with other forms of antitumor therapy including radiation and chemotherapy appears to offer stronger anticancer responses than either therapy alone [117]. These combinations, unfortunately, may also be associated with an increased frequency of toxicities and autoimmune reactions. Despite these challenges, multiple agents are being evaluated in different clinical trials. MEDI9197 is a dual agonist of TLR7/8 that is currently under phase I clinical test in combination with durvalumab and radiation therapy in metastatic or locally advanced solid malignancies (NCT02556463). Preliminary results demonstrate that the agent is overall safe with only mild adverse events. No efficacy data has been yet reported [118]. PG545 (pixatimod, pINN) is an agonist of TLR9/IL-12 that was tested in a phase I clinical trial in patients with advance solid tumors (NCT02042781). Results revealed that 3 out of 23 patients developed dose-limiting toxicities, and the disease control rate of 38% [119].

Polyinosinic-polycytidylic acid polylysine carboxymethylcellulose (poly-ICLC) is a potent TLR3 agonist that has been recently studied in combination with radiation in a phase I clinical trial in patients with HCC not eligible for surgery [120]. Intratumoral injection of this agent was found to be overall safe with mostly grade I or II adverse events. A PFS of 66% at 6 months and 28% at 24 months, OS of 69% after 1 year and 38% after 2 years were demonstrated [120].

### IL-2R

IL-2 mediates its immune-enhancing effect through either a low-affinity dimeric and/or a high-affinity trimeric IL-2 receptor (IL-2R). The dimeric IL-2R consists of CD122 (also known as IL-2Rβ) and CD132 (also known as ϒ_c_), whereas the trimeric IL-2R comprises an additional component, the CD25 (also known as IL-2Rα) which increases the affinity for its ligand [121].

IL-2 has been part of cancer treatment for many decades and is considered the first immunotherapy proven to be effective in human cancer in 1984 [121]. However, IL-2 has had certain limitations including a dual role enhancing both T cells and Tregs favoring immunosuppression, and a short life span with subsequent high doses requirements and potential severe toxicities including pulmonary edema, hypotension, and vascular leak syndrome [122]. In need of better strategies, IL-2R agonists have been developed to potentiate and prolong IL-2 antitumor effects allowing for lower doses and decreased toxicities [123]. Furthermore, IL-2R agonists could also enhance other forms of immunotherapy without the associated toxicity provided by IL-2.

NKTR-214, an engineered cytokine that specifically stimulates through CD122 (IL-2Rβ), is being tested in solid tumors including melanoma, NSCLC, and BC (NCT02869295, NCT02983045). Studies using both NKTR-214 and nivolumab showed no dose-limiting toxicities. One patient had a mixed radiographic response with a 40% decrease in LDH, and another patient had an unconfirmed CR after only 6 weeks of treatment [124]. Another trial showed no dose-limiting toxicities, a tumor shrinkage ranging from 10 to 30% in 6 out of 26 patients (23%) and an increase of T cells and NK cells within the tumor microenvironment in 100% of patients [125].

### Arginase inhibitors

Arginine is an important amino acid for T cell activation and proliferation. High levels of arginase are produced by malignant cells and MDSCs leading to depletion of arginine and a subsequent immunosuppressive tumor microenvironment [126]. The use of arginase inhibitors could allow overcoming the immunosuppressive effects of the tumor microenvironment and achieve a better antitumor control with the use of other immune checkpoint inhibitors or radiation therapy. Furthermore, the blockade of arginase may also have direct antitumor effects by decreasing the availability of substances that favor tumor growth [127]. Finally, given a higher expression of arginine among the tumor microenvironment than that in plasma, the use of these molecules could be associated with a more specific and less toxic effect than other forms of immunotherapy.

CB-1158 is a selective arginase inhibitor being studied in a phase I clinical trial alone or in combination with nivolumab in patients with metastatic solid tumors (NCT02903914). Preliminary results show that the drug is well tolerated with no dose-limiting toxicities, > 90% of arginase inhibition, and up to a fourfold increase in plasma arginine levels [128].

### Oncolytic peptides

Lactoferrin-derived lytic peptide LTX-315 is a cytotoxic chemotherapeutic peptide that permeabilizes mithocondrial membrane and triggers caspase-independent necrosis [129]. This agent modifies the tumor microenvironment by decreasing immunosuppressive cells and increasing T cells [130]. Intratumoral injection of this agent leads to tumor antigen release, with subsequent increase of TIL activity. This form of administration makes it an attractive way to limit systemic toxicities, but it also limits its applicability to more localized malignancies. Another important aspect of LTX-315 is the substantial increase of CTLA-4 expression following its administration. This suggests that this form of therapy may be particularly useful when used in conjunction with anti-CLTA-4 agents [131].

A phase I clinical trial using this molecule as monotherapy or in combination with ipilimumab or pembrolizumab is being conducted in patients with metastatic solid tumors, particularly melanoma and BC (NCT01986426). Preliminary results showed that 2/28 patients achieved a CR, 5 patients had a decreased of > 50% of tumor size, and 8 patients achieved SD [132].

### IL-10

IL-10 inhibits secretion of proinflammatory cytokines (e.g., IFNγ, TNFα, IL-1β, IL-6) and also inhibits the expression of MHC molecules and costimulatory molecules at several levels, leading to inhibition of T cell function [133]. Recently, IL-10 was also found to play some antitumor role by inducing the activation and proliferation of CD8. CD8 cells expressing IL-10 has been associated with a favorable prognosis in patients with lung cancer [134]. However, similar to other interleukins like IL-2, its effects are pleotropic and this raises concern for potential systemic toxicity. Other unresolved issues similar to IL-2 therapy include determining the patient population that could benefit the most from this form of therapy and the most appropriate therapeutic combinations [135]. In this regard, both PD-1 and IL-10 receptors are upregulated in TILs and therefore the combined use of these molecules is reasonable [136]. AM0010 is a PEGylated recombinant human IL-10 that is currently being studied in combination with pembrolizumab in melanoma patients in a phase I clinical trial (NCT02009449). Preliminary results revealed that 11 out of 25 recruited patients developed grade 3 or 4 adverse events including fatigue, thrombocytopenia, and anemia. Although no objective tumor response was seen, DCR was 45% [137].

### Limitations and challenges of immune checkpoint therapy

Although immune checkpoint therapy has been a great advancement in cancer treatment, several challenges such as immune-associated toxicity, treatment resistance, and clinical benefit limited to only a fraction of patients remain unresolved.

Immune checkpoint therapies are often associated with a set of toxicities known as immune-related adverse events, a form of autoimmune-like reactions resulting from an increased activity of the immune system. These toxicities can manifest as generalized symptoms including fatigue or fever, or can produce organ-specific damage leading to rash, colitis, pneumonitis, and adrenal or thyroid insufficiency, among many others [138–140]. Thus, using immune checkpoint therapy mandates a comprehensive understanding of these adverse events from clinicians as a way to prevent, recognize, and appropriately treat each specific reaction. Most adverse events are resolved with interruption of treatment and short course of steroids. Serious pneumonitis and colitis refractory of steroids may require use of biological agents, like infliximab [141–144].

Despite durable response rates observed with immune checkpoint therapy, the majority of patients do not benefit from the treatment (primary resistance), and some responders develop cancer progression after initial response (acquired resistance) [145]. Even within the same patient, heterogeneous responses have been observed in different metastatic lesions. Both tumor intrinsic and micro-environmental extrinsic factors contribute to this resistance. Tumor intrinsic mechanisms for resistance include the absence of tumor antigen, loss or downregulation of MHC, alteration of antigen presenting machinery such as beta-2 microglobulin mutation, alteration of pathways that prevent immune cell infiltration or function (mitogen-activated protein kinase, PI3K, WNT/b-catenin, Interferon-gamma pathways), and escape mutations in IFN signaling [145, 146]. Resistance can also be derived from extrinsic factors from tumor microenvironment. Regulatory T cells (Treg), MDSCs, M2 macrophages, and other inhibitory immune checkpoints may all contribute to inhibition of anti-tumor immune responses [147]. Understanding these mechanisms will assist with the process of designing new strategies to overcome resistance and provide the rationale for combination of different forms of immunotherapy [145, 147].

Clinical responses to checkpoint immunotherapy are variable. The identification of biomarkers to predict response and treatment-mediated toxicity remains an important unresolved issue. A number of biomarkers have been found promising. For example, immunohistochemical determination of PD-L1 expression, high mutational load, selective CD8+ T cell infiltration, and distribution at tumor invasive margins correlate with clinical response to anti-PD-1/PD-L1 treatment [148, 149]. A study demonstrated that the presence of epithelial-mesenchymal transition correlates with a distinct tumor microenvironment in lung cancer consisting of elevated inflammatory signals and multiple immune checkpoints [150]. Specific genes involved in chromatin remodeling may also serve as markers of response. As an example, the loss of function of the *PBRM1* gene encoding for the chromatin remodeling complex SWI/SNF was recently found to correlate with response to anti-PD-1 therapy in patients with clear cell RCC [151]. A recently developed model using malignancy-specific neoantigens appears to predict tumor response to ICI therapy in patients with melanoma and lung cancer receiving anti-CTLA-4 and anti-PD-1 therapy, respectively [152]. Furthermore, this model may also be useful to identify acquired resistance to therapy.

Lastly, immunotherapy is expensive and the cost per-quality life-year gained can be prohibitive in many developing countries, limiting its access to the eligible patients.

## Conclusions

Significant advances have been made in cancer immunotherapy in the last decade. Immune checkpoint therapy, particularly anti-CTLA4, anti-PD-1, or anti-PD-L1 antibodies, has revolutionized oncology care and quickly has become the standard of care in multiple malignancies. Immunotherapy targeting immune checkpoints is often better tolerated than traditional chemotherapy and durable responses are frequently seen. However, the clinical benefit has been limited to a subset of cancer patients. Furthermore, some who initially respond to treatment often relapse due to cancer resistance. Expanding clinical benefit to the majority of patients and preventing cancer resistance requires a better understanding of the mechanisms that lead to an effective anti-tumor response. The discovery of new immune inhibitory, stimulatory pathways, and rational combination strategies as discussed in this article will likely shed the light to the next step towards improvement of cancer immunotherapy.

## Abbreviations

A2aR: Adenosine A2a receptor
AACR: American Association for Cancer Research
APCs: Antigen presenting cells
ASCO: American Society of Clinical Oncology
B7-H3: B7 homolog 3
BC: Breast cancer
BTLA: B and T cell lymphocyte attenuator
CRC: Colorectal cancer
CRPC: Castrate-resistant prostate cancer
CTCL: Cutaneous T cell lymphomas
CTLA-4: Cytotoxic T lymphocyte-associated molecule-4
DART: Dual affinity re-targeting
DCR: Disease control rate
HCC: Hepatocellular carcinoma
HVEM: Herpes virus entry mediator
I-131: Radioactive iodine
ICIs: Immune checkpoint inhibitors
ICOS: Inducible co-stimulator
IDO: Indoleamine 2,3-dioxygenase
IFN: Interferon
IL: Interleukin
IL-2R: IL-2 receptor
KIRs: Killer immunoglobulin-like receptors
LAG-3: Lymphocyte activation gene-3
MDSCs: Myeloid-derived suppressor cells
MHC: Major Histocompatibility Complex
NK: Natural killer
NSCLC: Non-small cell lung carcinoma
OR: Objective responses
ORR: Objective response rate
PD-1: Programmed cell death receptor-1
PD-1H: Programmed death-1 homolog
PD-L1: Programmed cell death ligand-1
PFS: Progression-free survival
PI3Kγ: Phosphoinositide 3-kinase gamma
Poly-ICLC: Polyinosinic-polycytidylic acid polylysine carboxymethylcellulose
RCC: Renal cell carcinoma
SD: Stable disease
TGFβ: Transforming growth factor-β
TIGIT: T cell immunoglobulin and ITIM domain
TILs: Tumor-infiltrating lymphocytes
TIM-3: T cell immunoglobulin-3
TLRs: Toll-like receptors
VISTA: V-domain Ig suppressor of T cell activation
